# Real‐World Safety Profile of Spesolimab in Generalized Pustular Psoriasis: Insights From Japan as Part of a Multinational Expanded Access Program (EAP)

**DOI:** 10.1111/1346-8138.70122

**Published:** 2025-12-30

**Authors:** Akimichi Morita, Yayoi Tada, Yuichiro Tsunemi, Mayumi Komine, Takuro Kanekura, Shinichi Imafuku, Toshiya Takahashi, Xuemei Ding, Nichiren Pillai, Morihisa Saitoh, Rafael Sani Simoes, Nora Pöntynen, Keiichi Yamanaka

**Affiliations:** ^1^ Department of Geriatric and Environmental Dermatology Nagoya City University Graduate School of Medical Sciences Nagoya Japan; ^2^ Department of Dermatology Teikyo University School of Medicine Tokyo Japan; ^3^ Saitama Medical University Hospital Saitama Japan; ^4^ Jichi Medical University Hospital Tochigi Japan; ^5^ Department of Dermatology Kagoshima University Graduate School of Medical and Dental Sciences Kagoshima Japan; ^6^ Fukuoka University Hospital Fukuoka Japan; ^7^ Tohoku University Hospital Miyagi Japan; ^8^ Boehringer Ingelheim (China), Investment Co. Ltd Shanghai China; ^9^ Boehringer Ingelheim International Gmbh Ingelheim Am Rhein Germany; ^10^ Nippon Boehringer Ingelheim Co. Ltd Tokyo Japan; ^11^ Department of Dermatology Mie University Graduate School of Medicine Tsu Japan

**Keywords:** expanded access program, generalized pustular psoriasis, interleukin (IL)‐36, real‐world evidence, spesolimab

## Abstract

Generalized pustular psoriasis (GPP) is a heterogeneous, systemic neutrophilic inflammatory disease, characterized by chronic symptoms and recurrent flares, which can be potentially life‐threatening. Spesolimab, an interleukin‐36 receptor antagonist, has been approved to treat GPP flares in many countries including Japan. An expanded access program (EAP) in Japan provided early access to spesolimab for patients aged 18–75 years unable to participate in a clinical trial with no other treatment options. Patients received a single dose of 900 mg intravenous spesolimab for flare treatment, with an optional second dose after 1 week if symptoms persisted. Safety was monitored for 16 weeks post‐treatment. Eleven patients (54.5% female; 51 years mean age; 26.7 kg/m^2^ mean body mass index) received 23 doses of intravenous spesolimab. Nine patients (81.8%) were diagnosed with GPP for > 5 years. Ten patients (90.9%) had ≥ 1 baseline medical condition. All patients used ≥ 1 concomitant medication prior to or during the spesolimab treatment period, most commonly immunosuppressants and non‐steroidal anti‐inflammatory agents. Seven patients (63.6%) experienced treatment‐emergent adverse events, all of mild or moderate intensity, including skin and subcutaneous tissue disorders, general disorders and administration site conditions. No adverse event led to treatment discontinuation or death. A potential hypersensitivity event (face edema) resolved without intervention and was not considered treatment related. Spesolimab was well tolerated in a heterogeneous patient population with GPP, including those with comorbidities and concomitant medication use. The safety profile of spesolimab aligned with the EFFISAYIL 1 clinical trial results.

## Graphical abstract

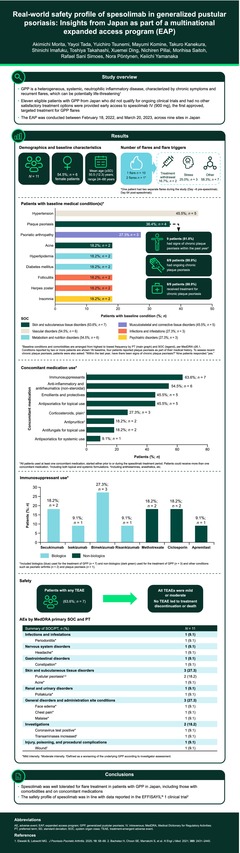

## 1 Introduction

GPP is a chronic, systemic, neutrophilic inflammatory disease [1, 2]. The disease course is heterogeneous and characterized by chronic symptoms and periods of flaring—rapid dissemination of painful skin manifestations, often accompanied by systemic symptoms [3, 4]. Chronic symptoms and periods of flaring greatly impact patients' quality of life [2, 4, 5]. GPP has a high comorbidity burden and can be life threatening [3, 6].

Spesolimab, a humanized monoclonal immunoglobulin G1 antibody that binds specifically to the interleukin (IL)‐36 receptor, is the first treatment targeting the IL‐36 pathway—a key GPP pathogenesis driver [7, 8, 9]. In the Phase II EFFISAYIL 1 study, a single 900 mg intravenous (IV) spesolimab dose led to rapid pustular and skin clearance within 1 week [10]. Based on the EFFISAYIL 1 results, spesolimab IV was approved for GPP flare treatment in countries including the USA, Europe, and Japan [7, 9, 10].

EAPs provide patients with severe or life‐threatening conditions access to therapies before regulatory approval [11]. They can also provide valuable real‐world evidence on treatment effectiveness and safety in more diverse representative patient populations than those in clinical trials [12]. In Japan, prior to regulatory spesolimab approval in September 2022 [7], an EAP provided access to spesolimab to patients with GPP presenting with a flare. We report descriptive safety and tolerability data from this EAP.

## 2 Methods

### 2.1 Study Design

The EAP was conducted across nine Japanese sites between February 18, 2022–March 20, 2023. Eligible patients aged 18–75 years were experiencing a flare, had no other satisfactory treatment options available (as assessed by the investigator), and were unable to participate in ongoing clinical trials (Table S1) [13]. Patients received a single IV spesolimab dose for flare treatment on Day 1, with an optional second dose 1 week later for persistent symptoms. Patients could re‐enter the EAP if they experienced a new GPP flare following spesolimab treatment after the 16‐week follow‐up period. Monitoring occurred at 1, 4, and 16 weeks after the last spesolimab dose (Figure S1).

### 2.2 Efficacy Assessments

In line with local regulations, the EAP did not assess efficacy. No disease activity or patient‐reported outcomes were collected.

### 2.3 Safety Assessments

Clinical laboratory evaluation was conducted for eligibility confirmation. Any additional laboratory testing during the trial was optional and performed at the discretion of the investigator.

Treatment‐emergent adverse events (TEAEs), treatment‐emergent serious adverse events (SAEs), and treatment‐emergent adverse events of special interest (AESIs) were collected during the 16‐week follow‐up period. Safety events were not stratified based on concomitant medication.

### 2.4 Consent

All patients provided written informed consent, in accordance with local regulatory and legal requirements.

## 3 Results

Eleven patients received spesolimab during the EAP: five males (45.5%) and six females (54.5%). The mean (± standard deviation) age was 50.5 ± 12.3 years. Over 80% of patients had GPP for ≥ 5 years before the EAP (Table 1). *IL36RN*, *CARD14*, and *AP1S3* mutations were reported as absent in four patients (36.4%) and unknown in seven patients (63.6%) for each mutation.

**TABLE 1:** Baseline demographics and characteristics.

	*N* = 11
Sex, *n* (%)	
Female	6 (54.5)
Male	5 (45.5)
Race, *n* (%)	
Asian	11 (100.0)
Age, years	
Mean ± SD	50.5 ± 12.3
Median (Q1, Q3)	56.0 (42.0, 57.0)
Range	24–68
BMI, kg/m^2^	
Mean ± SD	26.7 ± 6.8
Median (Q1, Q3)	25.5 (21.0, 29.7)
Time since diagnosis, *n* (%)	
≤ 1 year	2 (18.2)
> 1–≤ 5 years	0
> 5–≤ 10 years	3 (27.3)
> 10 years	6 (54.5)
Number of flares, *n* (%)	11 (100)
One flare	10 (90.9)
Two flares	1 (9.1)
Trigger event for flare, *n* (%)*	12 (100)
Treatment withdrawal	2 (16.7)
Stress	3 (25.0)
Other	7 (58.3)

Abbreviations: BMI, body mass index; SD, standard deviation.

* One patient had two recorded flare triggers.

Ten patients had a single flare (90.9%); nine of these patients received two spesolimab doses, and one patient received one dose. One patient (9.1%) had two separate flares during the study (Day −6 pre‐spesolimab, unknown precipitating event; Day 64 post‐spesolimab, precipitating event: decline in spesolimab efficacy), receiving a total of four spesolimab doses (two per flare). The patient had previously received prednisolone for GPP and continued to receive concomitant medications for GPP during the first treatment period, but was terminated before the second flare. All patients reported flare triggers (*n* = 12), with stress (25.0%; *n* = 3) and treatment withdrawal (16.7%; *n* = 2) being the most common. Other triggers were reported in 58.3% of patients: COVID‐19 vaccine (*n* = 1), lack of medication efficacy (*n* = 2), unknown (*n* = 4).

**FIGURE 1:**
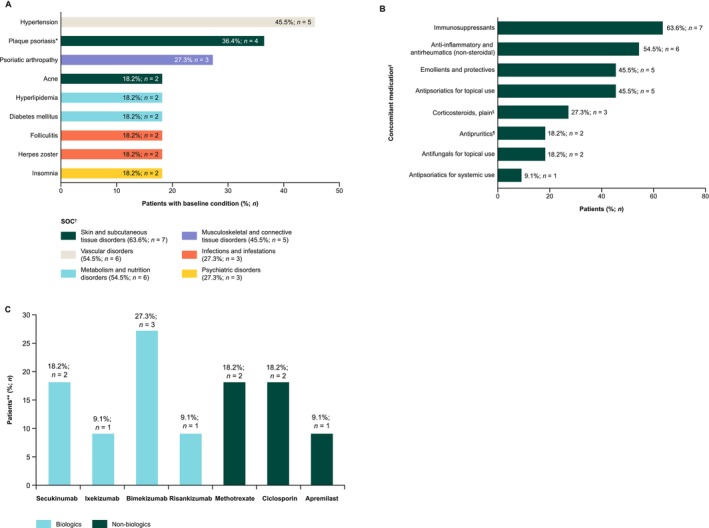
Overview of (A) most common baseline comorbidities per SOC and PT, (B) concomitant medications started either prior to or during the spesolimab treatment period, and (C) immunosuppressant use. *At baseline, four patients reported plaque psoriasis as a comorbidity as part of their medical history. Patients were also asked, “Within the last year, have there been signs of chronic plaque psoriasis?” (*n* = 9 responded “yes”). This more specific question was designed to record whether patients had recently active chronic plaque psoriasis. Among these nine patients, two patients reported severe, five reported moderate, and two reported mild chronic plaque psoriasis. Eight of the nine patients reported ongoing signs of chronic plaque psoriasis (data missing for one patient), and eight patients reported having received treatment. ^†^Baseline conditions and comorbidities are arranged from highest to lowest frequency by PT (main graph) and SOC (legend), per MedDRA v26.1. Conditions reported by two or more patients are shown in the graph ^‡^Selected concomitant medications started either prior to or during the spesolimab treatment period are shown. Some patients received more than one concomitant medication. ^§^Including both topical and systemic formulations ^¶^Including antihistamines, anesthetics, etc. **Including biologics (blue) used for the treatment of GPP (*n* = 7), and non‐biologics (dark green) used for the treatment of GPP (*n* = 3) and other conditions such as psoriatic arthritis (*n* = 2) and plaque psoriasis (*n* = 1). GPP, generalized pustular psoriasis; PT, preferred term; SOC, system organ class.

Most patients (90.9%; *n* = 10) had ≥ 1 baseline comorbidity. The most frequent system organ class was skin and subcutaneous tissue disorders (63.6%; *n* = 7); the most common preferred term (PT) was hypertension (45.5%; *n* = 5), followed by plaque psoriasis (36.4%; *n* = 4) and psoriatic arthropathy (27.3%; *n* = 3) (Figure 1A).

All patients used ≥ 1 concomitant medication, started prior to or during the spesolimab treatment period. Immunosuppressants (63.6%; *n* = 7) and non‐steroidal anti‐inflammatory and antirheumatic therapies (54.5%; *n* = 6) were the most commonly used (Figure 1B). Biologics used to treat GPP included the IL‐17 inhibitors bimekizumab (27.3%; *n* = 3), secukinumab (18.2%; *n* = 2), ixekizumab (9.1%; *n* = 1) and the IL‐23 inhibitor risankizumab (9.1%; *n* = 1). Non‐biologics included methotrexate (18.2%; *n* = 2) and apremilast (9.1%; *n* = 1) used to treat GPP, psoriatic arthritis, and/or plaque psoriasis; and ciclosporin (18.2%; *n* = 2) used to treat GPP (Figure 1C).

Seven patients (63.6%) experienced ≥ 1 TEAE, all of mild or moderate intensity. No TEAEs of severe or life‐threatening intensity, SAEs, or AESIs were reported. There were no investigator‐defined drug‐related adverse events (AEs). No AEs led to spesolimab discontinuation or death (Table 2). No AEs were reported based on vital signs. No patient experienced swelling, induration, heat, redness, or pain due to spesolimab.

**TABLE 2:** Overall summary of TEAEs and AEs by primary SOC and PT.

Summary of TEAEs, *n* (%)	*N* = 11
Patients with any AE	7 (63.6)
Patients with severe AEs (RCTC Grade 3 or 4)*	0
Patients with investigator‐defined, treatment‐related AEs	0
Patients with AEs leading to discontinuation of trial drug	0
Patients with investigator‐defined AESIs	0
Patients with serious AEs	0
SOC/PT, *n* (%)	***N* = 11**
Infections and infestations	**1 (9.1)**
Periodontitis^†^	1 (9.1)
Nervous system disorders	**1 (9.1)**
Headache^†^	1 (9.1)
Gastrointestinal disorders	**1 (9.1)**
Constipation^†^	1 (9.1)
Skin and subcutaneous tissue disorders	**3 (27.3)**
Pustular psoriasis^‡^ ^,^ ^§^	2 (18.2)
Acne^†^	1 (9.1)
Renal and urinary disorders	**1 (9.1)**
Pollakiuria^†^	1 (9.1)
General disorders and administration site conditions	**3 (27.3)**
Face edema^†^	1 (9.1)
Chest pain^†^	1 (9.1)
Malaise^†^	1 (9.1)
Investigations	**2 (18.2)**
Coronavirus test positive^†^	1 (9.1)
Transaminases increased^‡^	1 (9.1)
Injury, poisoning, and procedural complications	**1 (9.1)**
Wound^‡^	1 (9.1)

Abbreviations: AE, adverse event; AESI, adverse event of special interest; GPP, generalized pustular psoriasis; PT, preferred term; RCTC, Rheumatology Common Toxicity Criteria; SOC, system organ class.

* AE severity was graded using the RCTC v2.0.

^†^ Mild intensity.

^‡^ Moderate intensity.

^§^ Defined as a worsening of the underlying GPP according to investigator assessment. These two cases lasted 15 and 23 days, respectively.

Pustular psoriasis (investigator‐defined worsening) was the only TEAE reported in ≥ 1 patient (18.2%; *n* = 2). Both moderate cases resolved, with one patient requiring a medical intervention; neither event was considered drug‐related. General disorders (face edema, chest pain, malaise) were reported in three patients (*n* = 1 each). Other TEAEs reported at the MedDRA PT level included: single cases of periodontitis, headache, constipation, wound, pollakiuria. One patient (9.1%) experienced moderate, non‐treatment‐related transaminase elevation that required no intervention; another patient (9.1%) tested positive for COVID‐19. There were no respiratory‐ or cardiac‐related TEAEs. One patient experienced a potential hypersensitivity event (mild face edema) on Day 1, which resolved without intervention/complications on Day 50.

## 4 Discussion

This EAP provided early spesolimab access to 11 patients with GPP in Japan. Most patients had multiple comorbidities, including hyperlipidemia, diabetes mellitus, hypertension, and psoriatic arthropathy, in line with previous studies [6, 14]. Immunosuppressants, including biologics targeting the IL‐17 and IL‐23 pathways, were commonly used, with bimekizumab and secukinumab the most frequently used ones prior to or during spesolimab treatment. While these treatments are effective for plaque psoriasis, they may not sufficiently address GPP pathology, primarily driven by IL‐36 pathway dysregulation [8, 9]. Moreover, non‐steroidal anti‐inflammatory agents, topical antipsoriatics, emollients and protectives, and corticosteroids (topical and systemic) were commonly used, highlighting the significant symptom burden faced by these patients and the need to manage chronic GPP symptoms.

No treatment‐emergent SAEs or AESIs were reported. In addition, no AEs led to discontinuation of treatment or death. The most common TEAEs were mild to moderate, including skin reactions (worsening of pustular psoriasis) and general symptoms (malaise, chest pain). No AEs related to respiratory or thoracic disorders were reported. These findings align with safety findings from EFFISAYIL 1 [10, 15]. One patient (9.1%) experienced mild face edema on Day 1 post‐spesolimab, which resolved without intervention by Day 50. The event was not considered treatment‐related and may have been associated with underlying inflammation, suggested by its timing. The two cases of moderate worsening of underlying pustular psoriasis were not deemed treatment‐related; one patient's condition resolved without intervention, while the other required therapy.

Generalizability of the results is restricted by limited patient numbers and information, including genotyping, and no assessment of efficacy or laboratory monitoring. However, as the primary aim of an EAP is to provide treatment access rather than to assess efficacy, the lack of these measures is expected and not a study design limitation.

Spesolimab was well tolerated in a real‐world population of patients with GPP in Japan, including patients with co‐existing medical conditions and those taking concomitant medications, including other biologics. The safety profile of spesolimab aligns with the data reported in EFFISAYIL 1 [10].

## Funding

This work was supported by Boehringer Ingelheim.

## Ethics Statement

This EAP (NCT05200247) was carried out in compliance with the clinical trial protocol, in accordance with the principles of the Declaration of Helsinki, the International Conference on Harmonization Good Clinical Practice Guideline, Japanese Good Clinical Practice regulations (Ministry of Health and Welfare Ordinance No. 28, March 27, 1997), and other relevant regulations.

## Conflicts of Interest

A.M. has received research grants, consultancy fees, and/or speaker's fees from AbbVie, Amgen, Boehringer Ingelheim, Bristol Myers Squibb, Eli Lilly Japan, Janssen Pharmaceuticals, Kyowa Kirin, LEO Pharma, Maruho Co. Ltd., Sun Pharma Japan, Taiho Pharmaceutical, Torii Pharmaceutical, UCB Japan, and Ushio.

Y. Tada has received honoraria and/or grants from AbbVie, Boehringer Ingelheim, Eisai, Eli Lilly, Janssen, Kyowa Kirin, LEO Pharma, Maruho Co. Ltd., Mitsubishi Tanabe Pharma, Novartis, Sun Pharmaceutical Industries, Taiho Pharmaceutical, Torii Pharmaceutical, and UCB. Y. Tada is an Editorial Board member of The Journal of Dermatology and a co‐author of this article. To minimize bias, Y. Tada was excluded from all editorial decision‐making related to the acceptance of this article for publication.

Y. Tsunemi has received fees for lectures from Eli Lilly Japan K.K., Kyowa Kirin, Maruho Co. Ltd., Mitsubishi Tanabe Pharma Corporation, Novartis Pharma K.K., Sanofi K.K., Taiho Pharmaceutical, and Torii Pharmaceutical. Y. Tsunemi is an Editorial Board member of The *Journal of Dermatology* and a co‐author of this article. To minimize bias, Y. Tsunemi was excluded from all editorial decision‐making related to the acceptance of this article for publication.

M.K. has received speaker fees from Boehringer Ingelheim and research funds from Boehringer Ingelheim, Eli Lilly, Sun Pharma Japan, and Maruho Co. Ltd.

T.K. has no conflicts of interest.

S.I. has served as a consultant and/or paid speaker for and/or accepted a research grant from and/or participated in clinical trials sponsored by companies including AbbVie, Amgen, Boehringer Ingelheim, Bristol Myers Squibb, Eisai, Eli Lilly, Janssen, Kyowa Kirin, LEO Pharma, Maruho Co. Ltd., Mitsubishi Tanabe, Novartis, Pfizer, Sun Pharmaceutical Industries, Taiho Yakuhin Koryo, Torii Pharmaceutical, and UCB. S.I. is an Editorial Board member of The Journal of Dermatology and a co‐author of this article. To minimize bias, S.I. was excluded from all editorial decision‐making related to the acceptance of this article for publication.

T.T. has no conflicts of interest.

X.D., N.P., M.S., R.S.S., and N.P. are employees of Boehringer Ingelheim.

K.Y. has received research grants from AbbVie, Eisai, Eli Lilly, Kaken Pharmaceutical, Maruho Co. Ltd., Ministry of Education, Culture, Sports, Science and Technology, Japan, Japanese Society for Investigative Dermatology, Nihon Kayaku, Nihon Seiyaku, Sasaki Chemical, Sun Pharmaceutical Industries, Taiho Pharmaceutical, and Torii Pharmaceutical; and lecture and Chair fees from AbbVie, Boehringer Ingelheim, Celgene, Daiichi Sankyo, Eisai, Eli Lilly, Janssen, Kaken Pharmaceutical, Kyowa Kirin, LEO Pharma, Mitsubishi Tanabe, Maruho Co. Ltd., Nihon Kayaku, Novartis, Novel Pharma, Sanofi, Sun Pharmaceutical Industries, Taiho Pharmaceutical, Torii Pharmaceutical, and UCB.

## Supporting information


**Table S1:** Inclusion and exclusion criteria of EAP in Japan.
**Figure S1:** EAP study design, a new line with Plain Language Summary.

## Data Availability

The data that support the findings of this study are available from the corresponding author upon reasonable request.
