# Supplementary material for: Real‐World Safety Profile of Spesolimab in Generalized Pustular Psoriasis: Insights From Japan as Part of a Multinational Expanded Access Program (EAP)
Source: J Dermatol. 2025 Dec 30;53(2):351–6. doi: 10.1111/1346-8138.70122 (PMC12877984; doi:10.1111/1346-8138.70122)
Supplement: Supplementary file 1 — Table S1: Inclusion and exclusion criteria of EAP in Japan. Figure S1: EAP study design, a new line with Plain Language Summary. [file JDE-53-351-s001.docx]

**Supporting Information**

**Table S1. Inclusion and exclusion criteria of EAP in Japan**

| Inclusion criteria |
| --- |
| 1. Diagnosis of GPP confirmed based on the Japanese Dermatological Association guidelines for the management and treatment of GPP. |
| 1. Patient is experiencing a flare, defined as new or worsening of widespread eruption of sterile macroscopically visible pustules, with or without systemic inflammation, as assessed by the investigator. |
| 1. Male or female patients aged 18 to 75 years at time of enrollment. Women of childbearing potential must be willing and able to use a highly effective method of birth control per ICH M3 (R2) that results in a low failure rate of less than 1% per year when used consistently and correctly. A list of contraception methods meeting these criteria is provided in the patient information. |
| 1. Signed and dated written informed consent in accordance with ICH Good Clinical Practice and local legislation prior to admission to the trial. |
| 1. No satisfactory authorized alternative therapy exists, as assessed by the investigator. |
| Exclusion criteria |
| 1. Women who are pregnant, nursing, or who plan to become pregnant while in the trial.    1. Women who stop nursing before study drug administration do not need to be excluded from participating; they should refrain from breastfeeding for 16 weeks after the last spesolimab infusion. |
| 1. Severe, progressive, or uncontrolled hepatic disease, defined as >3-fold ULN elevation in AST or ALT or alkaline phosphatase, or >2-fold ULN elevation in total bilirubin. |
| 1. Active systemic infections (fungal and bacterial disease) during the last 2 weeks prior to drug administration, as assessed by the investigator. |
| 1. Increased risk of infectious complications (e.g. recent pyogenic infection, any congenital or acquired immunodeficiency [e.g. HIV], past organ or stem cell transplantation), as assessed by the investigator. |
| 1. Relevant chronic or acute infections, including active TB, HIV infection, or viral hepatitis at the time of drug administration.    1. Patients should be evaluated for TB infection prior to initiating treatment with spesolimab.    2. Anti-TB therapy should be considered, in accordance with local guidelines, prior to initiating spesolimab in patients with latent TB or a history of TB. |
| 1. History of allergy/hypersensitivity to systemically administered spesolimab or its excipients. |
| Exclusion criteria |
| 1. Any documented active or suspected malignancy or history of malignancy within 5 years prior to screening, except appropriately treated basal or squamous cell carcinoma of the skin or in situ carcinoma of uterine cervix. |
| 1. Immediate life-threatening flare of GPP requiring intensive care treatment according to the investigator’s judgment. Life-threatening complications include cardiovascular/cytokine-driven shock, pulmonary distress syndrome, or renal failure. |
| 1. Patients who must or wish to continue the intake of restricted medications (other IL-36R inhibitors, live vaccinations, or IL-1R/IL-1 inhibitors; see section 4.2.2.1) or any drug considered, in the judgment of the investigator, likely to interfere with the safe conduct of the study. |
| 1. Currently enrolled in another investigational device or drug study, or less than 30 days since ending another investigational device or drug study(s), or receiving other investigational treatment(s), or eligible to participate or participating in an ongoing actively accruing clinical trial with spesolimab in the treatment of GPP. |
| 1. A disease or condition that, in the opinion of the investigator, may put the patient at risk because of participation in this trial or limit the patient’s ability to participate in this trial. |
| 1. Presence of acute demyelinating neuropathy. |

ALT, alanine aminotransferase; AST, aspartate aminotransferase; GPP, generalized pustular psoriasis; HIV, human immunodeficiency virus; ICH, International Council for Harmonisation; IL, interleukin; TB, tuberculosis; ULN, upper limit of the normal range.

ClinicalTrials.gov. An expanded access trial in Japan to provide spesolimab to people with a flare-up in generalized pustular psoriasis who have no other treatment options. 2024. Available from: https://classic.clinicaltrials.gov/ct2/show/NCT05200247.

**Figure S1. EAP study design**


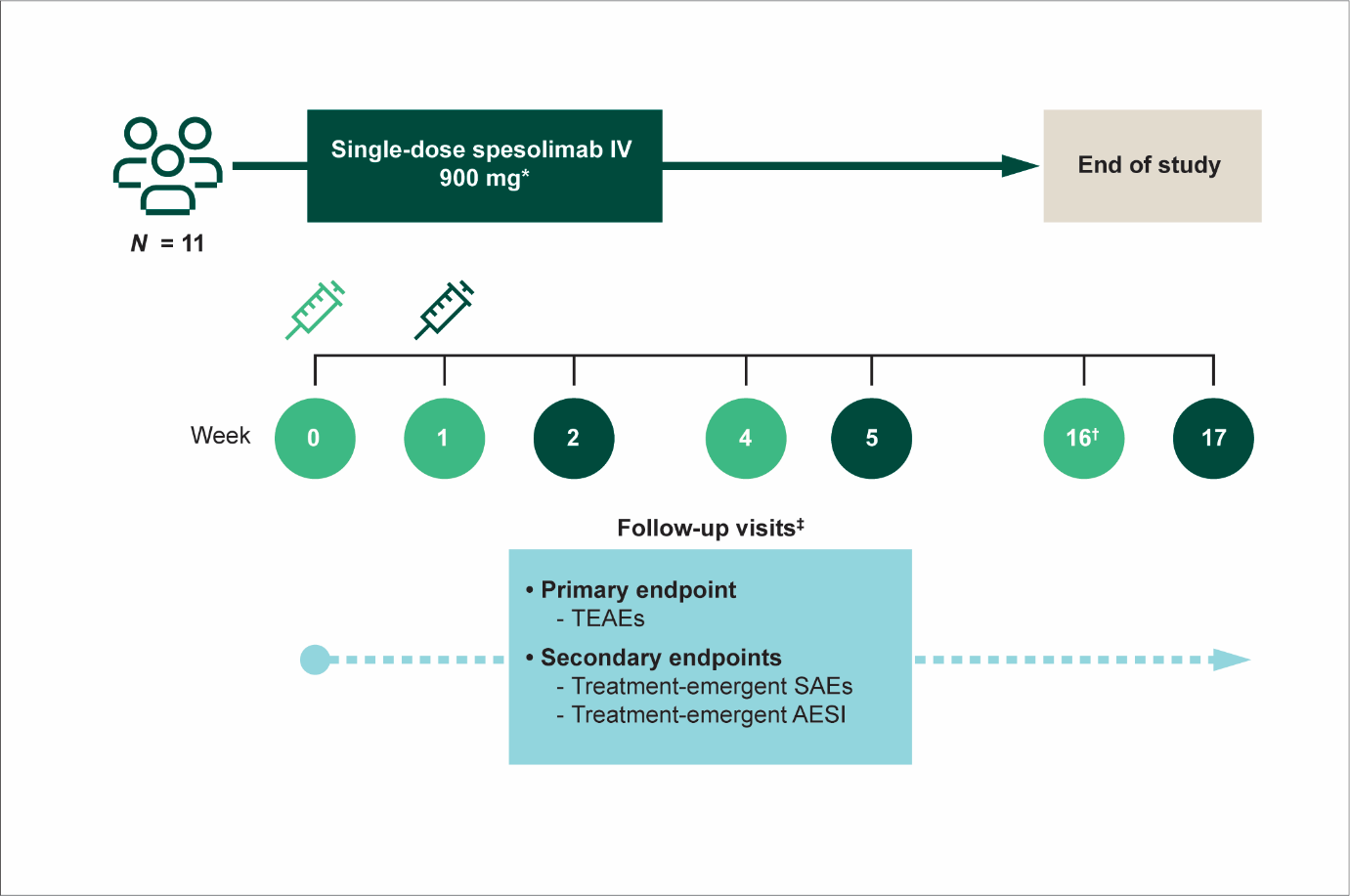


Each patient signed and dated an informed consent form according to local regulatory and legal requirements^.^

*Patients received a 900 mg dose of IV spesolimab for flare treatment, with an optional second dose after 1 week for persistent flare symptoms.

^†^If a patient experienced a new GPP flare following spesolimab treatment after the 16-week follow-up period, they could re-enter the trial and be treated again with spesolimab.

^‡^For patients who received one dose of spesolimab at Week 0, follow-up safety monitoring visits occurred at Week 1, Week 4, and Week 16 (green circles); for patients who received a second dose of spesolimab at Week 1, follow-up visits occurred at Week 2, Week 5, and Week 17 after the last dose of spesolimab (blue circles).

AE, adverse event; AESI, adverse event of special interest; EAP, expanded access program; GPP, generalized pustular psoriasis; IV, intravenous; SAE, serious adverse event; TEAE, treatment-emergent adverse event.

**Plain language summary**

Generalized pustular psoriasis (GPP) is a rare and life-long inflammatory disease that causes skin redness and sterile pustules (pus-filled bumps). People with GPP also often have skin pain, fever and fatigue (tiredness). The symptoms can suddenly become worse in episodes called flares, which can be life-threatening.

The EFFISAYIL^®^ 1 clinical study showed that a single dose (900 mg) of the drug spesolimab, which is administered intravenously (injected into a vein), rapidly cleared skin symptoms compared with placebo (non-active drug). Spesolimab has been approved to treat GPP flares in many countries, including Japan.

Expanded access programs (EAPs) give patients with severe medical conditions access to new drugs before they are approved. Between 2022 and 2023, an EAP in Japan offered spesolimab to patients with GPP for flare treatment. Patients could receive a second dose if their symptoms did not improve after 1 week. Safety information such as side effects was collected for 16 weeks after the last dose of spesolimab was given.

Eleven patients in Japan received intravenous spesolimab (all had at least one dose per flare). Most were diagnosed with GPP for more than 5 years and had comorbidities (other medical conditions). Seven patients experienced side effects, which were all mild or moderate. None of the patients needed to stop spesolimab treatment due to side effects. One patient reported face swelling, which doctors did not consider to be caused by spesolimab. Spesolimab was well tolerated, and the side effects were in line with those reported in the EFFISAYIL^®^ 1 study.
